# Composite Tests under Corrupted Data

**DOI:** 10.3390/e21010063

**Published:** 2019-01-14

**Authors:** Michel Broniatowski, Jana Jurečková, Ashok Kumar Moses, Emilie Miranda

**Affiliations:** 1 Laboratoire de Probabilités, Statistique et Modélisation, Sorbonne Université, 75005 Paris, France; 2 Institute of Information Theory and Automation, The Czech Academy of Sciences, 18208 Prague, Czech Republic; 3 Faculty of Mathematics and Physics, Charles University, 18207 Prague, Czech Republic; 4 Department of ECE, Indian Institute of Technology, Palakkad 560012, India; 5 Safran Aircraft Engines, 77550 Moissy-Cramayel, France

**Keywords:** composite hypotheses, corrupted data, least-favorable hypotheses, Neyman Pearson test, divergence based testing, Chernoff Stein lemma

## Abstract

This paper focuses on test procedures under corrupted data. We assume that the observations $Z_{i}$ are mismeasured, due to the presence of measurement errors. Thus, instead of $Z_{i}$ for i=1,…,n, we observe $X_{i}=Z_{i}+\sqrt{\delta }V_{i}$, with an unknown parameter δ and an unobservable random variable $V_{i}$. It is assumed that the random variables $Z_{i}$ are i.i.d., as are the $X_{i}$ and the $V_{i}$. The test procedure aims at deciding between two simple hyptheses pertaining to the density of the variable $Z_{i}$, namely $f_{0}$ and $g_{0}$. In this setting, the density of the $V_{i}$ is supposed to be known. The procedure which we propose aggregates likelihood ratios for a collection of values of δ. A new definition of least-favorable hypotheses for the aggregate family of tests is presented, and a relation with the Kullback-Leibler divergence between the sets ${\left(f_{\delta }\right)}_{\delta }$ and ${\left(g_{\delta }\right)}_{\delta }$ is presented. Finite-sample lower bounds for the power of these tests are presented, both through analytical inequalities and through simulation under the least-favorable hypotheses. Since no optimality holds for the aggregation of likelihood ratio tests, a similar procedure is proposed, replacing the individual likelihood ratio by some divergence based test statistics. It is shown and discussed that the resulting aggregated test may perform better than the aggregate likelihood ratio procedure.

## 1. Introduction

A situation which is commonly met in quality control is the following: Some characteristic *Z* of an item is supposed to be random, and a decision about its distribution has to be made based on a sample of such items, each with the same distribution $F_{0}$ (with density $f_{0}$) or $G_{0}$ (with density $g_{0}$). The measurement device adds a random noise $V_{\delta }$ to each measurement, mutually independent and independent of the item, with a common distribution function $H_{\delta }$ and density $h_{\delta }$, where δ is an unknown scaling parameter. Therefore the density of the measurement $X:=Z+V_{\delta }$ is either $f_{\delta }:=f_{0}*h_{\delta }$ or $g_{\delta }:=g_{0}\;*\;h_{\delta }$, where ∗ denotes the convolution operation. We denote $F_{\delta }$ (respectively $G_{\delta }$) to be the distribution function with density $f_{\delta }$ (respectively $g_{\delta }$).

The problem of interest, studied in [1], is how the measurement errors can affect the conclusion of the likelihood ratio test with statistics

$$L_{n}:=\frac{1}{n}\sum \log\frac{g_{0}}{f_{0}}\left(X_{i}\right).$$

For small δ, the result of [2] enables us to estimate the true log-likelihood ratio (true Kullback-Leibler divergence) even when we only dispose of locally perturbed data by additive measurement errors. The distribution function $H_{0}$ of the measurement errors is considered unknown, up to zero expectation and unit variance. When we use the likelihood ratio test, while ignoring the possible measurement errors, we can incur a loss in both errors of the first and second kind. However, it is shown, in [1], that for small δ the original likelihood ratio test (LRT) is still the most powerful, only on a slightly changed significance level. The test problem leads to a composite of null and alternative classes $H_{0}$ or $H_{1}$ of distributions of random variables $Z+V_{\delta }$ with $V_{\delta }:=\sqrt{\delta }V$, where *V* has distribution $H_{1}.$ If those families are bounded by alternating Choquet capacities of order 2, then the minimax test is based on the likelihood ratio of the pair of the least-favorable distributions of $H_{0}$ and $H_{1}$, respectively (see Huber and Strassen [3]). Moreover, Eguchi and Copas [4] showed that the overall loss of power caused by a misspecified alternative equals the Kullback-Leibler divergence between the original and the corrupted alternatives. Surprisingly, the value of the overall loss is independent of the choice of null hypothesis. The arguments of [2] and of [5] enable us to approximate the loss of power locally, for a broad set of alternatives. The asymptotic behavior of the loss of power of the test based on sampled data is considered in [1], and is supplemented with numerical illustration.

### Statement of the Test Problem

Our aim is to propose a class of statistics for testing the composite hypotheses $H_{0}$ and $H_{1}$, extending the optimal Neyman-Pearson LRT between $f_{0}$ and $g_{0}$. Unlike in [1], the scaling parameter δ is not supposed to be small, but merely to belong to some interval bounded away from 0.

We assume that the distribution *H* of the random variable (r.v.) *V* is known; indeed, in the tuning of the offset of a measurement device, it is customary to perform a large number of observations on the noise under a controlled environment.

Therefore, this first step produces a good basis for the modelling of the distribution of the density *h*. Although the distribution of *V* is known, under operational conditions the distribution of the noise is modified: For a given δ in $\left[\delta_{\min},\delta_{\max}\right]$ with $\delta_{\min}>0$, denote by $V_{\delta }$ a r.v. whose distribution is obtained through some transformation from the distribution of *V*, which quantifies the level of the random noise. A classical example is when $V_{\delta }=\sqrt{\delta }V$, but at times we have a weaker assumption, which amounts to some decomposability property with respect to δ: For instance, in the Gaussian case, we assume that for all δ,η, there exists some r.v. $W_{\delta ,\eta }$ such that $V_{\delta +\eta }{=}_{d}V_{\delta }+W_{\delta ,\eta }$, where $V_{\delta }$ and $W_{\delta ,\eta }$ are independent.

The test problem can be stated as follows: A batch of i.i.d. measurements $X_{i}:=Z_{i}+V_{\delta ,i}$ is performed, where δ>0 is unknown, and we consider the family of tests of $H_{0}$(δ):= [*X* has density $f_{\delta }$] vs. $H_{1}$(δ):= [*X* has density $g_{\delta }$], with $\delta \in \Delta =\left[\delta_{\min},\delta_{\max}\right].$ Only the $X_{i}$ are observed. A class of combined tests of $H_{0}$ vs. $H_{1}$ is proposed, in the spirit of [6,7,8,9].

Under every fixed *n*, we assume that δ is allowed to run over a finite set $p_{n}$ of components of the vector $\Delta_{n}:=\left[\delta_{\min}=\delta_{0,n},\ldots ,\delta_{p_{n},n}=\delta_{\max}\right]$. The present construction is essentially non-asymptotic, neither on *n* nor on δ, in contrast with [1], where δ was supposed to lie in a small neighborhood of 0. However, with increasing *n*, it would be useful to consider that the array ${\left(\delta_{j,n}\right)}_{j=1}^{p_{n}}$ is getting dense in $\Delta =\left[\delta_{\min},\delta_{\max}\right]$ and that

(1) $$\lim_{n\to \infty }\frac{\log p_{n}}{n}=0.$$

For the sake of notational brevity, we denote by Δ the above grid $\Delta_{n}$, and all suprema or infima over Δ are supposed to be over $\Delta_{n}$. For any event *B* and any δ in Δ, $F_{\delta }\left(B\right)$ (respectively $G_{\delta }\left(B\right)$) designates the probability of *B* under the distribution $F_{\delta }$ (respectively $G_{\delta }$). Given a sequence of levels $\alpha_{n}$, we consider a sequence of test criteria $T_{n}:=T_{n}\left(X_{1},\ldots ,X_{n}\right)$ of $H_{0}\left(\delta \right)$, and the pertaining critical regions

(2) $$T_{n}\left(X_{1},\ldots ,X_{n}\right)>A_{n},$$

such that

$$F_{\delta }\left(T_{n}\left(X_{1},\ldots ,X_{n}\right)>A_{n}\right)\leq \alpha_{n}\;\forall \delta \in \Delta ,$$

leading to rejection of $H_{0}$(δ) for at least some δ∈Δ.

In an asymptotic context, it is natural to assume that $\alpha_{n}$ converges to 0 as *n* increases, since an increase in the sample size allows for a smaller first kind risk. For example, in [8], $\alpha_{n}$ takes the form $\alpha_{n}:=\exp\left\{-na_{n}\right\}$ for some sequence $a_{n}\to \infty$.

In the sequel, the Kullback-Leibler discrepancy between probability measures *Q* and *P*, with respective densities *p* and *q* (with respect to the Lebesgue measure on R), is denoted

$$K\left(Q,P\right):=\int \log\frac{q(x)}{p(x)}q\left(x\right)dx$$

whenever defined, and takes value +∞ otherwise.

The present paper handles some issues with respect to this context. In Section 2, we consider some test procedures based on the supremum of Likelihood Ratios (LR) for various values of δ, and define $T_{n}$. The threshold for such a test is obtained for any level $\alpha_{n}$, and a lower bound for its power is provided. In Section 3, we develop an asymptotic approach to the Least Favorable Hypotheses (LFH) for these tests. We prove that asymptotically least-favorable hypotheses are obtained through minimization of the Kullback-Leibler divergence between the two composite classes H0 and H1 independently upon the level of the test.

We next consider, in Section 3.3, the performance of the test numerically; indeed, under the least-favorable pair of hypotheses we compare the power of the test (as obtained through simulation) with the theoretical lower bound, as obtained in Section 2. We show that the minimal power, as measured under the LFH, is indeed larger than the theoretical lower bound—this result shows that the simulation results overperform on theoretical bounds. These results are developed in a number of examples.

Since no argument plays in favor of any type of optimality for the test based on the supremum of likelihood ratios for composite testing, we consider substituting those ratios with other kinds of scores in the family of divergence-based concepts, extending the likelihood ratio in a natural way. Such an approach has a long history, stemming from the seminal book by Liese and Vajda [10]. Extensions of the Kullback-Leibler based criterions (such as the likelihood ratio) to power-type criterions have been proposed for many applications in Physics and in Statistics (see, e.g., [11]). We explore the properties of those new tests under the pair of hypotheses minimizing the Kullback-Leibler divergence between the two composite classes H0 and H1. We show that, in some cases, we can build a test procedure whose properties overperform the above supremum of the LRTs, and we provide an explanation for this fact. This is the scope of Section 4.

## 2. An Extension of the Likelihood Ratio Test

For any δ in Δ, let

(3) $$T_{n,\delta }:=\frac{1}{n}\sum_{i=1}^{n}\log\frac{g_{\delta }}{f_{\delta }}\left(X_{i}\right),\;$$

and define

$$T_{n}:=\underset{\delta \in \Delta }{\sup}T_{n,\delta }.$$

Consider, for fixed δ, the Likelihood Ratio Test with statistics $T_{n,\delta }$ which is uniformly most powerful (UMP) within all tests of $H0(\delta ):=p_{T}=f_{\delta }$ vs. $H1(\delta ):=p_{T}=g_{\delta }$, where $p_{T}$ designates the distribution of the generic r.v. *X*. The test procedure to be discussed aims at solving the question: Does there exist some δ, for which H0(δ) would be rejected vs. H1(δ), for some prescribed value of the first kind risk?

Whenever H0(δ) is rejected in favor of H1(δ), for some δ, we reject $H0:=f_{0}=g_{0}$ in favor of $H1:=f_{0}\neq g_{0}$. A critical region for this test with level $\alpha_{n}$ is defined by

$$T_{n}>A_{n},$$

with

$$\begin{array}{lll} P_{H0}\left(H1\right) & = & \underset{\delta \in \Delta }{\sup}F_{\delta }\left(T_{n}>A_{n}\right)\\ \; & = & \underset{\delta \in \Delta }{\sup}F_{\delta }\left(\underset{\delta^{\prime }}{⋃}T_{n,\delta^{\prime }}>A_{n}\right)\leq \alpha_{n}. \end{array}$$

Since, for any sequence of events $B_{1},\ldots ,B_{p_{n}}$,

$$F_{\delta }\left(\overset{p_{n}}{\underset{k=1}{⋃}}B_{k}\right)\leq p_{n}\max_{1\leq k\leq p_{n}}F_{\delta }\left(B_{k}\right),$$

it holds that

(4) $$P_{H0}\left(H1\right)\leq p_{n}\max_{\delta \in \Delta }\max_{\delta^{\prime }\in \Delta }F_{\delta }\left(T_{n,\delta^{\prime }}>A_{n}\right).$$

An upper bound for $P_{H0}\left(H1\right)$ can be obtained, making use of the Chernoff inequality for the right side of (4), providing an upper bound for the risk of first kind for a given $A_{n}$. The correspondence between $A_{n}$ and this risk allows us to define the threshold $A_{n}$ accordingly.

Turning to the power of this test, we define the risk of second kind by

(5) $$\begin{array}{ll} P_{H_{1}}\left(H0\right) & :=\underset{\eta \in \Delta }{\sup}G_{\eta }\left(T_{n}\leq A_{n}\right)\\ \; & =\underset{\eta \in \Delta }{\sup}G_{\eta }\left(\underset{\delta \in \Delta }{\sup}T_{n,\delta }\leq A_{n}\right)\\ \; & =\underset{\eta \in \Delta }{\sup}G_{\eta }\left(\underset{\delta \in \Delta }{⋂}T_{n,\delta }\leq A_{n}\right)\\ \; & \leq \underset{\eta \in \Delta }{\sup}G_{\eta }\left(T_{n,\eta }\leq A_{n}\right), \end{array}$$

a crude bound which, in turn, can be bounded from above through the Chernoff inequality, which yields a lower bound for the power of the test under any hypothesis $g_{\eta }$ in H1.

Let $\alpha_{n}$ denote a sequence of levels, such that

$$\lim\underset{n\to \infty }{\sup}\alpha_{n}<1.$$

We make use of the following hypothesis:

(6) $$\underset{\delta \in \Delta }{\sup}\underset{\delta^{\prime }\in \Delta }{\sup}\int \log\frac{f_{\delta^{\prime }}}{g_{\delta^{\prime }}}f_{\delta }<0.$$

**Remark** **1.** *Since*

$$\int \log\frac{f_{\delta^{\prime }}}{g_{\delta^{\prime }}}f_{\delta }=K\left(F_{\delta },G_{\delta^{\prime }}\right)-K\left(F_{\delta },F_{\delta^{\prime }}\right),$$

*making use of the Chernoff-Stein Lemma (see Theorem A1 in the Appendix A), Hypothesis (6) means that any LRT with H0: $p_{T}=f_{\delta }$ vs. H1: $p_{T}=g_{\delta^{\prime }}$ is asymptotically more powerful than any LRT with H0: $p_{T}=f_{\delta }$ vs. H1: $p_{T}=f_{\delta^{\prime }}.$*

Both hypotheses (7) and (8), which are defined below, are used to provide the critical region and the power of the test.

For all $\delta ,\delta^{\prime }$ define

$$Z_{\delta^{\prime }}:=\log\frac{g_{\delta^{\prime }}}{f_{\delta^{\prime }}}\left(X\right),$$

and let

$$\varphi_{\delta ,\delta^{\prime }}\left(t\right):=\log E_{F_{\delta }}\left(\exp\left(tZ_{\delta^{\prime }}\right)\right)=\log\int {\left(\frac{g_{\delta^{\prime }}\left(x\right)}{f_{\delta^{\prime }}\left(x\right)}\right)}^{t}f_{\delta }\left(x\right)dx.$$

With $N_{\delta ,\delta^{\prime }}$, the set of all *t* such that $\varphi_{\delta ,\delta^{\prime }}\left(t\right)$ is finite, we assume

(7) $$N_{\delta ,\delta^{\prime }}\;\mathrm{is}\;a\;\mathrm{non}\;\mathrm{void}\;\mathrm{open}\;\mathrm{neighborhood}\;\mathrm{of}\;0.$$

Define, further,

$$J_{\delta ,\delta^{\prime }}\left(x\right):=\underset{t}{\sup}tx-\varphi_{\delta ,\delta^{\prime }}\left(t\right),$$

and let

$$J\left(x\right):=\min_{\left(\delta ,\delta^{\prime }\right)\in \Delta \times \Delta }J_{\delta ,\delta^{\prime }}\left(x\right).$$

For any η, let

$$W_{\eta }:=-\log\frac{g_{\eta }}{f_{\eta }}\left(X\right),$$

and let

$$\psi_{\eta }\left(t\right):=\log E_{G_{\eta }}\left(\exp\left(tW_{\eta }\right)\right).$$

Let $M_{\eta }$ be the set of all *t* such that $\psi_{\eta }\left(t\right)$ is finite. Assume

(8) $$M_{\eta }\;\mathrm{is}\;a\;\mathrm{non}\;\mathrm{void}\;\mathrm{neighborhood}\;\mathrm{of}\;0.$$

Let

(9) $$I_{\eta }\left(x\right):=\underset{t}{\sup}tx-\log E_{G_{\eta }}\left(\exp\left(tW_{\eta }\right)\right),$$

and

$$I\left(x\right):=\underset{\eta }{\inf}I_{\eta }\left(x\right).$$

We also assume an accessory condition on the support of $Z_{\delta^{\prime }}$ and $W_{\eta }$, respectively under $F_{\delta }$ and under $G_{\eta }$ (see (A2) and (A5) in the proof of Theorem A1). Suppose the regularity assumptions (7) and (8) are fulfilled for all $\delta ,\delta^{\prime }$ and η. Assume, further, that $p_{n}$ fulfills (1).

The following result holds:

**Proposition** **2.** *Whenever (6) holds, for any sequence of levels $\alpha_{n}$ bounded away from* 1, *defining*

$$A_{n}:=J^{-1}\left(-\frac{1}{n}\log\frac{\alpha_{n}}{p_{n}}\right),$$

*it holds, for large n, that*

$$\;P_{H0}\left(H1\right)=\underset{\delta \in \Delta }{\sup}F_{\delta }\left(T_{n}>A_{n}\right)\leq \alpha_{n}$$

*and*

$$P_{H1}\left(H1\right)=\underset{\delta \in \Delta }{\sup}G_{\delta }\left(T_{n}>A_{n}\right)\geq 1-\exp\left(-nI\left(A_{n}\right)\right).$$

## 3. Minimax Tests under Noisy Data, Least-Favorable Hypotheses

### 3.1. An Asymptotic Definition for the Least-Favorable Hypotheses

We prove that the above procedure is asymptotically minimax for testing the composite hypothesis $H_{0}$ against the composite alternative $H_{1}$. Indeed, we identify the least-favorable hypotheses, say $F_{\delta_{*}}\in H_{0}$ and $G_{\delta_{*}}\in H_{1}$, which lead to minimal power and maximal first kind risk for these tests. This requires a discussion of the definition and existence of such a least-favourable pair of hypotheses in an asymptotic context; indeed, for a fixed sample size, the usual definition only leads to an explicit definition in very specific cases. Unlike in [1], the minimax tests will not be in the sense of Huber and Strassen. Indeed, on one hand, hypotheses $H_{0}$ and $H_{1}$ are not defined in topological neighbourhoods of $F_{0}$ and $G_{0}$, but rather through a convolution under a parametric setting. On the other hand, the specific test of $\left\{H_{0}\left(\delta \right),\;\delta \in \Delta \right\}$ against $\left\{H_{1}\left(\delta \right),\;\delta \in \Delta \right\}$ does not require capacities dominating the corresponding probability measures.

Throughout the subsequent text, we shall assume that there exists $\delta_{*}$ such that

(10) $$\min_{\delta \in \Delta }K\left(F_{\delta },G_{\delta }\right)=K\left(F_{\delta_{*}},G_{\delta_{*}}\right).$$

We shall call the pair of distributions $\left(F_{\underline{\delta }},\;G_{\underline{\delta }}\right)$ least-favorable for the sequence of tests $1\left\{T_{n}>A_{n}\right\}$ if they satisfy

(11) $$\begin{array}{cc} F_{\delta }\left(T_{n}\leq A_{n}\right)\geq F_{\underline{\delta }}\left(T_{n}\leq A_{n}\right)\\ \;\geq G_{\underline{\delta }}\left(T_{n}\leq A_{n}\right)\geq G_{\delta }\left(T_{n}\leq A_{n}\right) & \end{array}$$

for all δ∈Δ. The condition of unbiasedness of the test is captured by the central inequality in (11).

Because, for finite *n*, such a pair can be constructed only in few cases, we should take a recourse of (11) to the asymptotics n→∞. We shall show that any pair of distributions $\left(F_{\delta_{*}}G_{\delta_{*}}\right)$ achieving (10) are least-favorable. Indeed, it satisfies the inequality (11) asymptotically on the logarithmic scale.

Specifically, we say that $\left(F_{\underline{\delta }},G_{\underline{\delta }}\right)$ is a least-favorable pair of distributions when, for any δ∈Δ,

(12) $$\begin{array}{cc} \lim\underset{n\to \infty }{\inf}\frac{1}{n}\log F_{\underline{\delta }}\left(T_{n}\leq A_{n}\right) & \geq \lim_{n\to \infty }\frac{1}{n}\log G_{\underline{\delta }}\left(T_{n}\leq A_{n}\right)\\ \; & \geq \lim_{n\to \infty }\sup\frac{1}{n}\log G_{\delta }\left(T_{n}\leq A_{n}\right). \end{array}$$

Define the total variation distance

$$d_{TV}\left(F_{\delta },G_{\delta }\right):=\underset{B}{\sup}\left|F_{\delta }\left(B\right)-G_{\delta }\left(B\right)\right|,$$

where the supremum is over all Borel sets *B* of R. We will assume that, for all *n*,

(13) $$\alpha_{n}<1-\underset{\delta \in \Delta }{\sup}d_{TV}\left(F_{\delta },G_{\delta }\right).$$

We state our main result, whose proof is deferred to the Appendix B.

**Theorem** **3.** *For any level $\alpha_{n}$ satisfying (13), the pair $\left(F_{\delta_{*}},G_{\delta_{*}}\right)$ is a least-favorable pair of hypotheses for the family of tests $1\left\{T_{n}\geq A_{n}\right\}$, in the sense of (12).*

### 3.2. Identifying the Least-Favorable Hypotheses

We now concentrate on (10).

For given $\delta \in \left[\delta_{\min},\delta_{\max}\right]$ with $\delta_{\min}>0$, the distribution of the r.v. $V_{\delta }$ is obtained through some transformation from the known distribution of V. The classical example is $V_{\delta }=\sqrt{\delta }V$, which is a scaling, and where $\sqrt{\delta }$ is the signal to noise ratio. The following results state that the Kullback-Leibler discrepancy $K\left(F_{\delta },G_{\delta }\right)$ reaches its minimal value when the noise $V_{\delta }$ is “maximal”, under some additivity property with respect to δ. This result is not surprising: Adding noise deteriorates the ability to discriminate between the two distributions $F_{0}$ and $G_{0}$—this effect is captured in $K\left(F_{\delta },G_{\delta }\right)$, which takes its minimal value for the maximal δ.

**Proposition** **4.** *Assume that, for all δ,η, there exists some r.v. $W_{\delta ,\eta }$ such that $V_{\delta +\eta }{=}_{d}V_{\delta }+W_{\delta ,\eta }$ where $V_{\delta }$ and $W_{\delta ,\eta }$ are independent. Then*

$$\delta_{*}=\delta_{\max}.$$

This result holds as a consequence of Lemma A5 in the Appendix C.

In the Gaussian case, when *h* is the standard normal density, Proposition 4 holds, since $h_{\delta +\eta }=h_{\delta }*h_{\eta -\delta }$ with $h_{\varepsilon }\left(x\right):=\left(1/\sqrt{\varepsilon }\right)h\left(x/\sqrt{\varepsilon }\right).$ In order to model symmetric noise, we may consider a symmetrized Gamma density as follows: Set $h_{\delta }\left(x\right):=\left(1/2\right)\gamma^{+}\left(1,\delta \right)\left(x\right)+\left(1/2\right)\gamma^{-}\left(1,\delta \right)\left(x\right)$, where $\gamma^{+}\left(1,\delta \right)$ designates the Gamma density with scale parameter 1 and shape parameter δ, and $\gamma^{-}\left(1,\delta \right)$ the Gamma density on $R^{-}$ with same parameter. Hence a r.v. with density $h_{\delta }$ is symmetrically distributed and has variance 2δ. Clearly, $h_{\delta +\eta }\left(x\right)=h_{\delta }*h_{\eta }\left(x\right)$, which shows that Proposition 4 also holds in this case. Note that, except for values of δ less than or equal to 1, the density $h_{\delta }$ is bimodal, which does not play in favour of such densities for modelling the uncertainty, due to the noise. In contrast with the Gaussian case, $h_{\delta }$ cannot be obtained from $h_{1}$ by any scaling. The centred Cauchy distribution may help as a description of heavy tailed symmetric noise, and keeps uni-modality through convolution; it satisfies the requirements of Proposition 4 since $f_{\delta }*f_{\eta }\left(x\right)=f_{\delta +\eta }\left(x\right)$ where $f_{\varepsilon }\left(x\right):=\varepsilon /\pi \left(x^{2}+\varepsilon^{2}\right)$. In this case, δ acts as a scaling, since $f_{\delta }$ is the density of δX where *X* has density $f_{1}.$

In practice, the interesting case is when δ is the variance of the noise and corresponds to a scaling of a generic density, as occurs for the Gaussian case or for the Cauchy case. In the examples, which will be used below, we also consider symmetric, exponentially distributed densities (Laplace densities) or symmetric Weibull densities with a given shape parameter. The Weibull distribution also fulfills the condition in Proposition 4, being infinitely divisible (see [12]).

### 3.3. Numerical Performances of the Minimax Test

As frequently observed, numerical results deduced from theoretical bounds are of poor interest due to the sub-optimality of the involved inequalities. They may be sharpened on specific cases. This motivates the need for simulation. We study two cases, which can be considered as benchmarks.

- In the first case, $f_{0}$ is a normal density with expectation 0 and variance 1, whereas $g_{0}$ is a normal density with expectation 0.3 and variance 1.
- The second case handles a situation where $f_{0}$ and $g_{0}$ belong to different models: $f_{0}$ is a log-normal density with location parameter 1 and scale parameter 0.2, whereas $g_{0}$ is a Weibull density on $R^{+}$ with shape parameter 5 and scale parameter 3. Those two densities differ strongly, in terms of asymptotic decay. They are, however, very close to one another in terms of their symmetrized Kullback-Leibler divergence (the so-called Jeffrey distance). Indeed, centering on the log-normal distribution $f_{0}$, the closest among all Weibull densities is at distance 0.10—the density $g_{0\;}$ is at distance 0.12 from $f_{0}.$

Both cases are treated, considering four types of distribution for the noise:

- The noise $h_{\delta }$ is a centered normal density with variance $\delta^{2}$;
- the noise $h_{\delta }$ is a centered Laplace density with parameter λ(δ);
- the noise $h_{\delta }$ is a symmetrized Weibull density with shape parameter 1.5 and variable scale parameter β(δ); and
- the noise $h_{\delta }$ is Cauchy with density $h_{\delta }\left(x\right)=\gamma \left(\delta \right)/\pi (\gamma {\left(\delta \right)}^{2}+x^{2})$.

In order to compare the performances of the test under those four distributions, we have adopted the following rule: The parameter of the distribution of the noise is tuned such that, for each value $\underline{\delta }$, it holds that $P\left(\left|V_{\underline{\delta }}\right|>\underline{\delta }\right)=\Phi \left(1\right)-\Phi \left(-1\right)∼0.65$, where Φ stands for the standard Gaussian cumulative function. Thus, distributions b to d are scaled with respect to the Gaussian noise with variance $\delta^{2}$.

In both cases A and B, the range of δ is $\Delta =\left(\delta_{\min}=0.1,\delta_{\max}\right)$, and we have selected a number of possibilities for $\delta_{\max}$, ranging from 0.2 to 0.7.

In case A, we selected $=\delta_{\max}^{2}=0.5$, which has a signal-to-noise ratio equal to 0.7, a commonly chosen bound in quality control tests.

In case B, the variance of $f_{0}$ is roughly 0.6 and the variance of $g_{0}$ is roughly 0.4. The maximal value of $\delta_{\max}^{2}$ is roughly 0.5. This is thus a maximal upper bound for a practical modeling.

We present some power functions, making use of the theoretical bounds together with the corresponding ones based on simulation runs. As seen, the performances in the theoretical approach is weak. We have focused on simulation, after some comparison with the theoretical bounds.

#### 3.3.1. Case A: The Shift Problem

In this subsection, we evaluate the quality of the theoretical power bound, defined in the previous sections. Thus, we compare the theoretical formula to the empirical lower performances obtained through simulations under the least-favorable hypotheses.

#### Theoretical Power Bound

While supposedly valid for finite *n*, the theoretical power bound given by (A8) still assumes some sort of asymptotics, since a good approximation of the bound implies a fine discretization of Δ to compute $I\left(A_{n}\right)=\inf_{\eta \in \Delta_{n}}I_{\eta }\left(A_{n}\right)$. Thus, by condition (1), *n* has to be large. Therefore, in the following, we will compute this lower bound for *n* sufficiently large (that is, at least 100 observations), which is also consistent with industrial applications.

#### Numerical Power Bound

In order to obtain a minimal bound for the power of the composite test, we compute the power of the test $H_{0}\left(\delta_{*}\right)$ against $H_{1}\left(\delta_{*}\right)$, where $\delta_{*}$ defines the LFH pair $\left(F_{\delta_{*}},G_{\delta_{*}}\right)$.

Following Proposition 4, the LFH for the test defined by $T_{n}$ when the noise follows a Gaussian, a Cauchy, or a symmetrized Weibull distribution is achieved for $\left(F_{\delta_{\max}},G_{\delta_{\max}}\right)$.

When the noise follows a Laplace distribution, the pair of LFH is the one that satisfies:

(14) $$\left(F_{\delta_{*}},G_{\delta_{*}}\right)=\arg\min_{\left(F_{\delta },G_{\delta }\right),\delta \in \Delta_{n}}K\left(F_{\delta },G_{\delta }\right).$$

In both of the cases A and B, this condition is also satisfied for $\delta^{*}=\delta_{\max}$.

#### Comparison of the Two Power Curves

**Figure 1:**
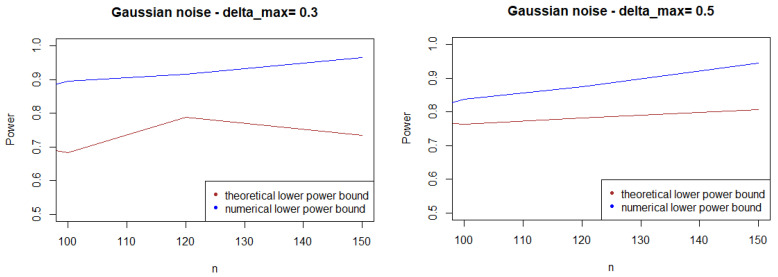
Theoretical and numerical power bound of the test of case A under Gaussian noise (with respect to *n*), for the first kind risk α=0.05.

**Figure 2:**
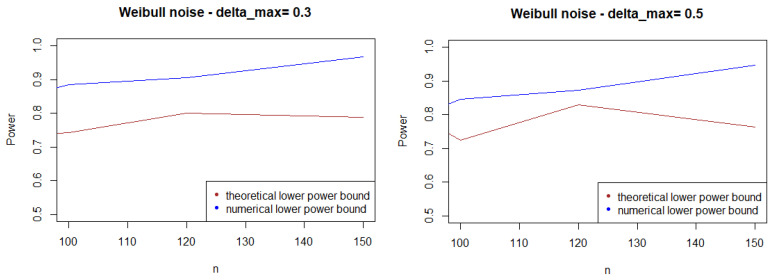
Theoretical and numerical power bound of the test of case A under symmetrized Weibull noise (with respect to *n*), for the first kind risk α=0.05.

**Figure 3:**
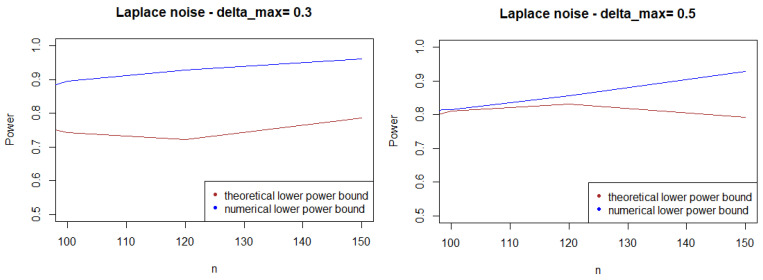
Theoretical and numerical power bound of the test of case A under a symmetrized Laplacian noise (with respect to *n*), for the first kind risk α=0.05.

As expected, Figure 1, Figure 2 and Figure 3 show that the theoretical lower bound is always below the empirical lower bound, when *n* is high enough to provide a good approximation of $I(A_{n})$. This is also true when the noise follows a Cauchy distribution, but for a bigger sample size than in the figures above (n>250).

In most cases, the theoretical bound tends to largely underestimate the power of the test, when compared to its minimal performance given by simulations under the least-favorable hypotheses. The gap between the two also tends to increase as *n* grows. This result may be explained by the large bound provided by (5), while the numerical performances are obtained with respect to the least-favorable hypotheses.

From a computational perspective, the computational cost of the theoretical bound is far higher than its numeric counterpart.

#### 3.3.2. Case B: The Tail Thickness Problem

The calculation of the moment-generating function, appearing in the formula of $I_{\eta }\left(x\right)$ in (9), is numerically unstable, which renders the computation of the theoretical bound impossible. Thus, in the following sections, the performances of the test will be evaluated numerically, through Monte Carlo replications.

## 4. Some Alternative Statistics for Testing

### 4.1. A Family of Composite Tests Based on Divergence Distances

This section provides a similar treatment as above, dealing now with some extensions of the LRT test to the same composite setting. The class of tests is related to the divergence-based approach to testing, and it includes the cases considered so far. For reasons developed in Section 3.3, we argue through simulation and do not develop the corresponding large deviation approach.

The statistics $T_{n}$ can be generalized in a natural way, by defining a family of tests depending on some parameter γ. For γ≠0,1, let

$$\phi_{\gamma }\left(x\right):=\frac{x^{\gamma }-\gamma x+\gamma -1}{\gamma (\gamma -1)}$$

be a function defined on $\left(0,\infty \right)$ with values in $\left(0,\infty \right)$, setting

$$\phi_{0}\left(x\right):=-\log x+x-1$$

and

$$\phi_{1}\left(x\right):=x\log x-x+1.$$

For γ≤2, this class of functions is instrumental in order to define the so-called power divergences between probability measures, a class of pseudo-distances widely used in statistical inference (see, for example, [13]).

Associated to this class, consider the function

$$\begin{array}{cc} \varphi_{\gamma }\left(x\right) & :=-\frac{d}{dx}\phi_{\gamma }\left(x\right)\\ \; & =\frac{1-x^{\gamma -1}}{\gamma -1}\;\mathrm{for}\;\gamma \neq 0,1. \end{array}$$

**Figure 4:**
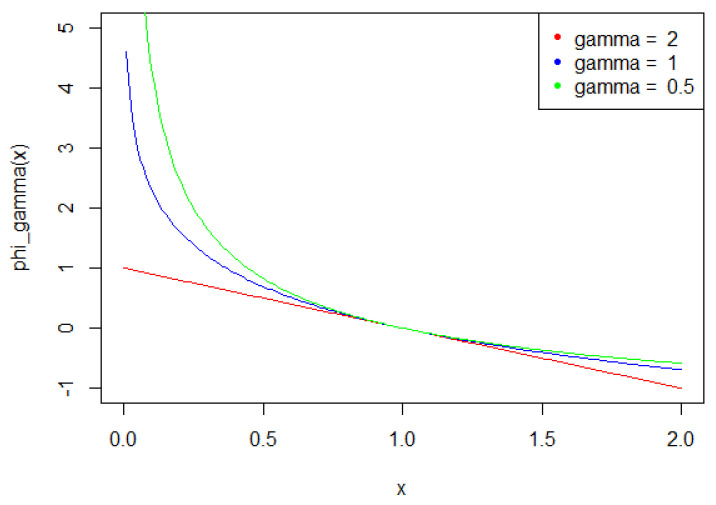
$\varphi_{\gamma }$ for γ=0.5,1, and 2.

We also consider

$$\begin{array}{l} \varphi_{1}\left(x\right):=-\log x\\ \varphi_{0}\left(x\right):=\frac{1}{x}-1, \end{array}$$

from which the statistics

$$T_{n,\delta }^{\gamma }:=\frac{1}{n}\sum_{i=1}^{n}\varphi_{\gamma }\left(X_{i}\right)$$

and

$$T_{n}^{\gamma }:=\underset{\delta }{\sup}T_{n,\delta }^{\gamma }$$

are well defined, for all γ≤2. Figure 4 illustrates the functions $\varphi_{\gamma }$, according to γ.

Fix a risk of first kind α, and the corresponding power of the LRT pertaining to $H0(\delta_{*})$ vs. $H1(\delta_{*})$ by

$$1-\beta :=G_{\delta_{*}}\left(T_{n,\delta_{*}}^{1}>s_{\alpha }\right),$$

with

$$s_{\alpha }:=\inf\left\{s:F_{\delta_{*}}\left(T_{n,\delta_{*}}^{1}>s\right)\leq \alpha \right\}.$$

Define, accordingly, the power of the test, based on $T_{n}^{\gamma }$ under the same hypotheses, by

$$s_{\alpha }^{\gamma }:=\inf\left\{s:F_{\delta_{*}}\left(T_{n}^{\gamma }>s\right)\leq \alpha \right\}$$

and

$$1-\beta^{\prime }:=G_{\delta_{*}}\left(T_{n}^{\gamma }>s_{\alpha }^{\gamma }\right).$$

First, $\delta_{*}$ defines the pair of hypotheses $\left(F_{\delta_{*}},G_{\delta_{*}}\right)$, such that the LRT with statistics $T_{n,\delta_{*}}^{1}$ has maximal power among all tests $H0(\delta_{*})$ vs. $H1(\delta_{*}).$ Furthermore, by Theorem A1, it has minimal power on the logarithmic scale among all tests H0(δ) vs. H1(δ).

On the other hand, $\left(F_{\delta_{*}},G_{\delta_{*}}\right)$ is the LF pair for the test with statistics $T_{n}^{1}$ among all pairs $\left(F_{\delta },G_{\delta }\right).$

These two facts allow for the definition of the loss of power, making use of $T_{n}^{1}$ instead of $T_{n,\delta_{*}}^{1}$ for testing $H0(\delta_{*})$ vs. $H1(\delta_{*}).$ This amounts to considering the price of aggregating the local tests $T_{n,\delta }^{1}$, a necessity since the true value of δ is unknown. A natural indicator for this loss consists in the difference

$$\Delta_{n}^{1}:=G_{\delta_{*}}\left(T_{n,\delta_{*}}^{1}>s_{\alpha }\right)-G_{\delta_{*}}(T_{n}^{1}>s_{\alpha }^{1})\geq 0.$$

Consider, now, aggregated test statistics $T_{n}^{\gamma }.$ We do not have at hand a similar result, as in Proposition 2. We, thus, consider the behavior of the test $H0(\delta_{*})$ vs. $H1(\delta_{*})$, although $\left(F_{\delta_{*}},G_{\delta_{*}}\right)$ may not be a LFH for the test statistics $T_{n}^{\gamma }.$ The heuristics, which we propose, makes use of the corresponding loss of power with respect to the LRT, through

$$\Delta_{n}^{\gamma }:=G_{\delta_{*}}\left(T_{n,\delta_{*}}^{1}>s_{\alpha }\right)-G_{\delta_{*}}\left(T_{n}^{\gamma }>s_{\alpha }^{\gamma }\right).$$

We will see that it may happen that $\Delta_{n}^{\gamma }$ improves over $\Delta_{n}^{1}.$ We define the optimal value of γ, say $\gamma^{*}$, such that

$$\Delta_{n}^{\gamma^{*}}\leq \Delta_{n}^{\gamma },$$

for all γ.

In the various figures hereafter, NP corresponds to the LRT defined between the LFH’s $\left(F_{\delta_{*}},G_{\delta_{*}}\right),$ KL to the test with statistics $T_{n}^{1\;}$ (hence, as presented Section 2), HELL corresponds to $T_{n}^{1/2\;}$, which is associated to the Hellinger power divergence, and G = 2 corresponds to γ=2.

### 4.2. A Practical Choice for Composite Tests Based on Simulation

We consider the same cases A and B, as described in Section 3.3.

As stated in the previous section, the performances of the different test statistics are compared, considering the test of $H_{0}\left(\delta_{*}\right)$ against $H_{1}\left(\delta_{*}\right)$ where $\delta^{*}$ is defined, as explained in Section 3.3 as the LFH for the test $T_{n}^{1}$. In both cases A and B, this corresponds to $\delta^{*}=\delta_{\max}$.

#### 4.2.1. Case A: The Shift Problem

Overall, the aggregated tests perform well, when the problem consists in identifying a shift in a distribution. Indeed, for the three values of γ (0.5, 1, and 2), the power remains above 0.7 for any kind of noise and any value of $\delta_{*}$. Moreover, the power curves associated to $T_{n}^{\gamma }$ mainly overlap with the optimal test $T_{n,\delta_{*}}^{1}$.

**Figure 5:**
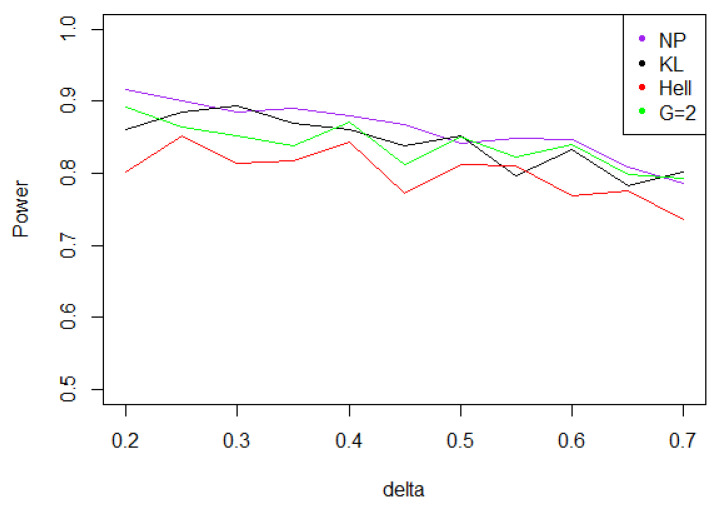
Power of the test of case A under Gaussian noise (with respect to $\delta_{\max}$), for the first kind risk α=0.05 and sample size n=100.

**Figure 6:**
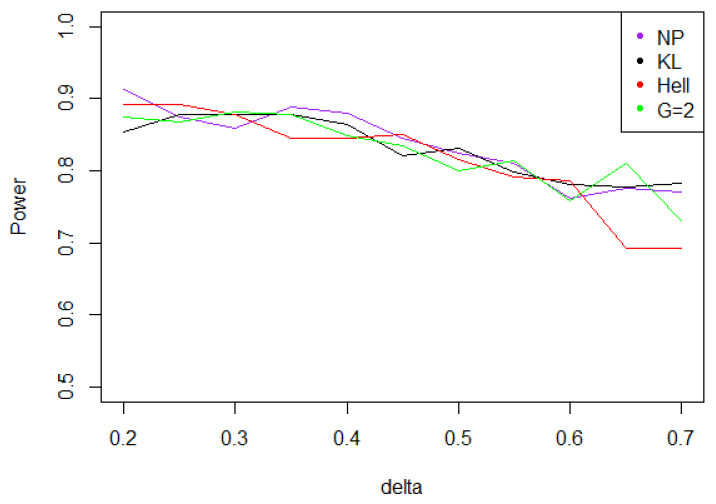
Power of the test of case A under Laplacian noise (with respect to $\delta_{\max}$), for the first kind risk α=0.05 and sample size n=100.

**Figure 7:**
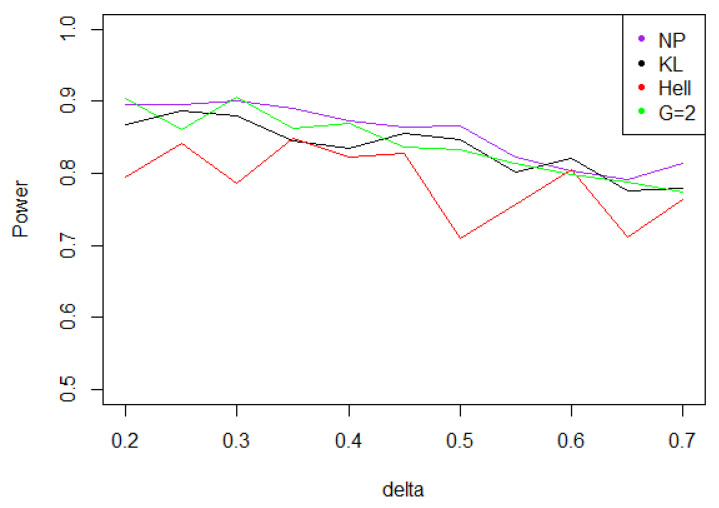
Power of the test of case A under symmetrized Weibull noise (with respect to $\delta_{\max}$), for the first kind risk α=0.05 and sample size n=100.

**Figure 8:**
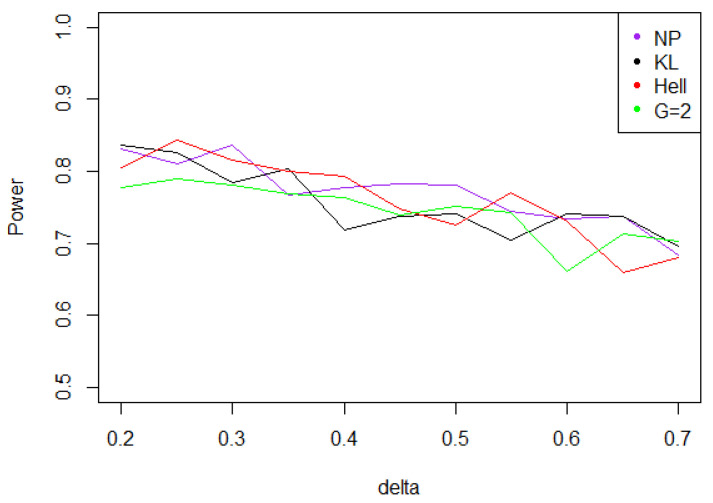
Power of the test of case A under noise following a Cauchy distribution (with respect to $\delta_{\max}$), for the first kind risk α=0.05 and sample size n=100.

- Under Gaussian noise, the power remains mostly stable over the values of $\delta_{*}$, as shown by Figure 5. The tests with statistics $T_{n}^{1}$ and $T_{n}^{2}$ are equivalently powerful for large values of $\delta_{*}$, while the first one achieves higher power when $\delta_{*}$ is small.
- When the noise follows a Laplace distribution, the three power curves overlap the NP power curve, and the different test statistics can be indifferently used. Under such a noise, the alternative hypotheses are extremely well distinguished by the class of tests considered, and this remains true as $\delta_{*}$ increases (cf. Figure 6).
- Under the Weibull hypothesis, $T_{n}^{1}$ and $T_{n}^{2}$ perform similarly well, and almost always as well as $T_{n,\delta_{*}}^{1}$, while the power curve associated to $T_{n}^{1/2}$ remains below. Figure 7 illustrates that, as $\delta_{\max}$ increases, the power does not decrease much.
- Under a Cauchy assumption, the alternate hypotheses are less distinguishable than under any other parametric hypothesis on the noise, since the maximal power is about 0.84, while it exceeds 0.9 in cases a, b, and c (cf. Figure 5, Figure 6, Figure 7 and Figure 8). The capacity of the tests to discriminate between $H0(\delta_{\max})$ and $H1(\delta_{\max})$ is almost independent of the value of $\delta_{\max}$, and the power curves are mainly flat.

#### 4.2.2. Case B: The Tail Thickness Problem

**Figure 9:**
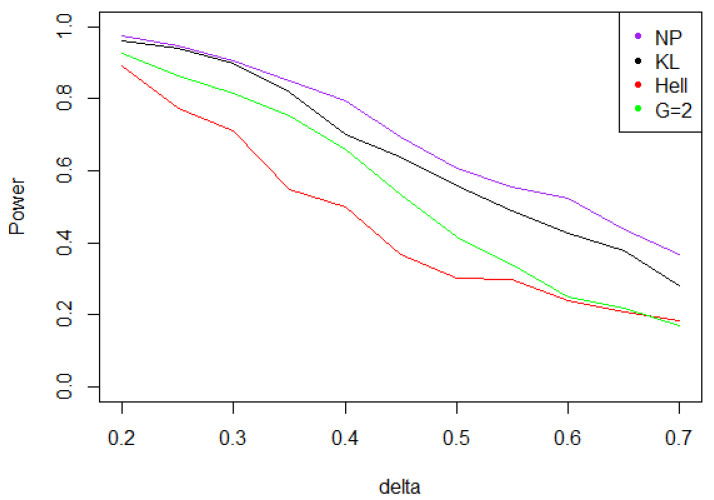
Power of the test of case B under Gaussian noise (with respect to $\delta_{\max}$), for the first kind risk α=0.05 and sample size n=100. The NP curve corresponds to the optimal Neyman Pearson test under $\delta_{\max}$. The KL, Hellinger, and G=2 curves stand respectively for γ=1,γ=0.5, and γ=2 cases.

**Figure 10:**
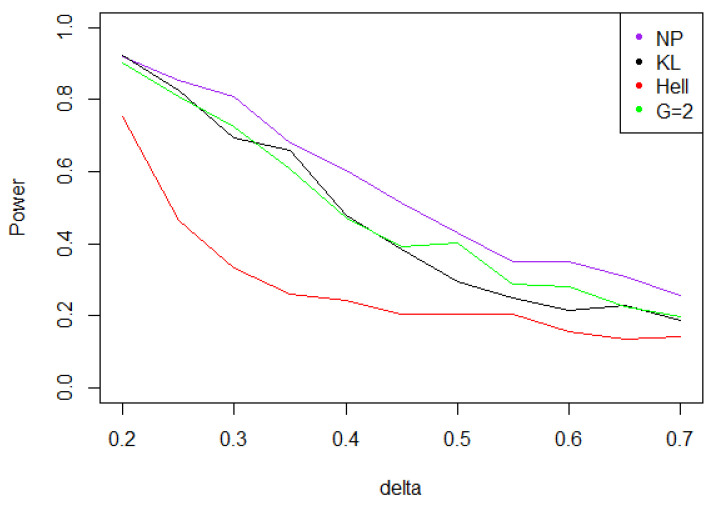
Power of the test of case B under Laplacian noise (with respect to $\delta_{\max}$), for the first kind risk α=0.05 and sample size n=100.

**Figure 11:**
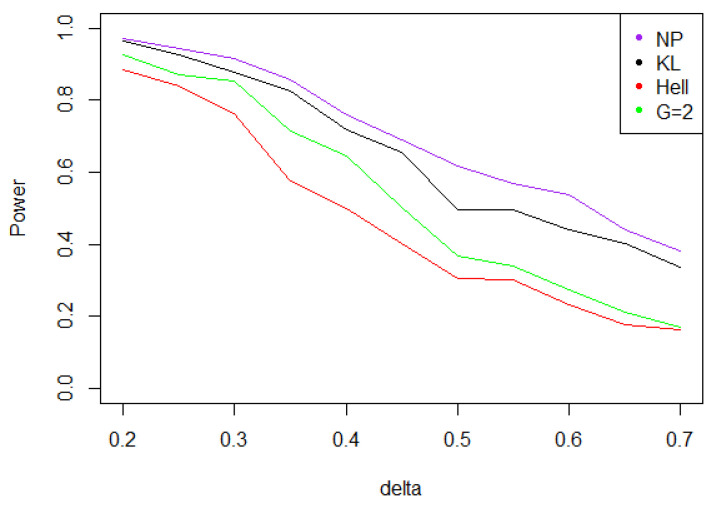
Power of the test of case B under symmetrized Weibull noise (with respect to $\delta_{\max}$), for the first kind risk α=0.05 and sample size n=100.

**Figure 12:**
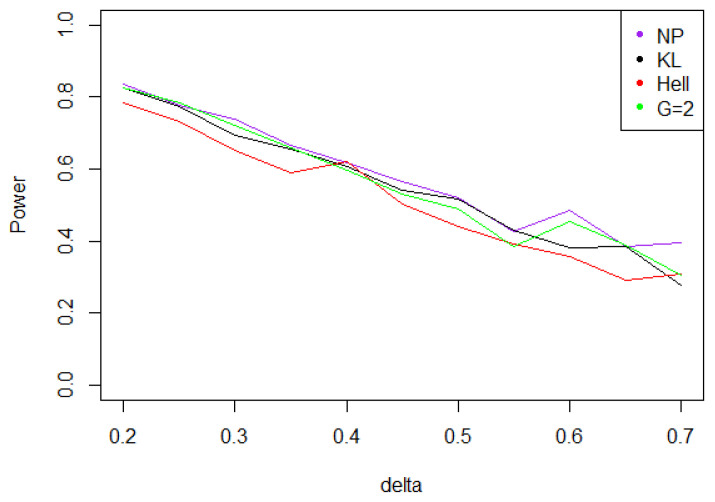
Power of the test of case B under a noise following a Cauchy distribution (with respect to $\delta_{\max}$), for the first kind risk α=0.05 and sample size n=100.

- With the noise defined by case A (Gaussian noise), for KL (γ=1), $\delta_{*}=\delta_{\max}$ due to Proposition 4 and statistics $T_{n}^{1}$ provides the best power uniformly upon $\delta_{\max}.$ Figure 9 shows a net decrease of the power as $\delta_{\max}$ increases (recall that the power is evaluated under the least favorable alternative $G_{\delta_{\max}}$).
- When the noise follows a Laplace distribution, the situation is quite peculiar. For any value of δ in Δ, the modes $M_{G_{\delta_{\max}}}\;$ and $M_{F_{\delta_{\max}}}$ of the distributions of $\left(f_{\delta }/g_{\delta }\right)\left(X\right)$ under $G_{\delta_{\max}}$ and under $F_{\delta_{\max}}$ are quite separated; both larger than 1. Also, for δ all the values of $\left|\phi_{\gamma }(M_{G_{\delta_{\max}}})-\phi_{\gamma }(M_{F_{\delta_{\max}}})\right|$ are quite large for large values of γ. We may infer that the distributions of $\phi_{\gamma }\left(\left(f_{\delta }/g_{\delta }\right)\left(X\right)\right)$ under $G_{\delta_{\max}}$ and under $F_{\delta_{\max}}$ are quite distinct for all δ, which in turn implies that the same fact holds for the distributions of $T_{n}^{\gamma }$ for large γ. Indeed, simulations presented in Figure 10 show that the maximal power of the test tends to be achieved when γ=2.
- When the noise follows a symmetric Weibull distribution, the power function when γ=1 is very close to the power of the LRT between $F_{\delta_{\max}}$ and $G_{\delta_{\max}}$ (cf. Figure 11). Indeed, uniformly on δ, and on *x*, the ratio $\left(f_{\delta }/g_{\delta }\right)\left(x\right)$ is close to 1. Therefore, the distribution of $T_{n}^{1}$ is close to that of $T_{n,\delta_{\max}}^{1}$, which plays in favor of the KL composite test.
- Under a Cauchy distribution, similarly to case A, Figure 12 shows that $T_{n}^{\gamma }$ achieves the maximal power for γ=1 and 2, closely followed by γ=0.5.

## 5. Conclusions

We have considered a composite testing problem, where simple hypotheses in either H0 and H1 were paired, due to corruption in the data. The test statistics were defined through aggregation of simple likelihood ratio tests. The critical region for this test and a lower bound of its power was produced. We have shown that this test is minimax, evidencing the least-favorable hypotheses. We have considered the minimal power of the test under such a least favorable hypothesis, both theoretically and by simulation, and for a number of cases (including corruption by Gaussian, Laplacian, symmetrized Weibull, and Cauchy noise). Whatever this noise, the actual minimal power, as measured through simulation, was quite higher than obtained through analytic developments. Least-favorable hypotheses were defined in an asymptotic sense, and were proved to be the pair of simple hypotheses in H0 and H1 which are closest, in terms of the Kullback-Leibler divergence; this holds as a consequence of the Chernoff-Stein Lemma. We, next, considered aggregation of tests where the likelihood ratio was substituted by a divergence-based statistics. This choice extended the former one, and may produce aggregate tests with higher power than obtained through aggregation of the LRTs, as examplified and analysed. Open questions are related to possible extensions of the Chernoff-Stein Lemma for divergence-based statistics.

## Appendix A. Proof of Proposition 2

### Appendix A.1. The Critical Region of the Test

Define

$$Z_{\delta^{\prime }}:=\log\frac{g_{\delta^{\prime }}}{f_{\delta^{\prime }}}\left(X\right),$$

which satisfies

$$\begin{array}{cc} E_{F_{\delta }}\left(Z_{\delta^{\prime }}\right) & =\int \log\frac{g_{\delta^{\prime }}}{f_{\delta^{\prime }}}\left(x\right)f_{\delta }\left(x\right)dx\\ \; & =\int \log\frac{g_{\delta^{\prime }}}{f_{\delta }}\left(x\right)f_{\delta }\left(x\right)dx+\int \log\frac{f_{\delta }}{f_{\delta^{\prime }}}\left(x\right)f_{\delta }\left(x\right)dx\\ \; & =K\left(F_{\delta },G_{\delta^{\prime }}\right)-K\left(F_{\delta },G_{\delta^{\prime }}\right). \end{array}$$

Note that, for all δ,

$$K\left(F_{\delta },G_{\delta^{\prime }}\right)-K\left(F_{\delta },G_{\delta^{\prime }}\right)=\int \log\frac{g_{\delta^{\prime }}}{f_{\delta^{\prime }}}f_{\delta }$$

is negative for $\delta^{\prime }$ close to δ, assuming that

$$\delta^{\prime }\mapsto \int \log\frac{g_{\delta^{\prime }}}{f_{\delta^{\prime }}}f_{\delta }$$

is a continuous mapping. Assume, therefore, that (6) holds, which means that the classes of distributions $\left(G_{\delta }\right)$ and $\left(F_{\delta }\right)$ are somehow well separated. This implies that $E_{F_{\delta }}\left(Z_{\delta^{\prime }}\right)<0$, for all δ and $\delta^{\prime }.$

In order to obtain an upper bound for $F_{\delta }\left(T_{n,\delta^{\prime }}\left(X_{n}\right)>A_{n}\right)$, for all $\delta ,\delta^{\prime }$ in Δ, through the Chernoff Inequality, consider

$$\varphi_{\delta ,\delta^{\prime }}\left(t\right):=\log E_{F_{\delta }}\left(\exp\left(tZ_{\delta^{\prime }}\right)\right)=\log\int {\left(\frac{g_{\delta^{\prime }}\left(x\right)}{f_{\delta^{\prime }}\left(x\right)}\right)}^{t}g_{\delta }\left(x\right)dx.$$

Let

$$\;t^{+}\left(N_{\delta ,\delta^{\prime }}\right):=\sup\left\{t\in N_{\delta ,\delta^{\prime }}:\varphi_{\delta ,\delta^{\prime }}\left(t\right)<\infty \right\}.$$

The function $\left(\delta ,\delta^{\prime },x\right)\mapsto J_{\delta ,\delta^{\prime }}\left(x\right)$ is continuous on its domain, and since $t\mapsto \varphi_{\delta ,\delta^{\prime }}\left(t\right)$ is a strictly convex function which tends to infinity as *t* tends to $t^{+}\left(N_{\delta ,\delta^{\prime }}\right)$, it holds that

$$\lim_{x\to \infty }J_{\delta ,\delta^{\prime }}\left(x\right)=+\infty$$

for all $\delta ,\delta^{\prime }$ in $\Delta_{n}.$

We now consider an upper bound for the risk of first kind on a logarithmic scale.

We consider

(A1) $$A_{n}>E_{F_{\delta }}\left(Z_{\delta^{\prime }}\right),$$

for all $\delta ,\delta^{\prime }$. Then, by the Chernoff inequality

$$\frac{1}{n}\log F_{\delta }\left(T_{n,\delta^{\prime }}\left(X_{n}\right)>A_{n}\right)\leq -J_{\delta ,\delta^{\prime }}\left(A_{n}\right).$$

Since $A_{n}$ should satisfy

$$\exp\left(-nJ_{\delta ,\delta^{\prime }}\left(A_{n}\right)\right)\leq \alpha_{n},$$

with $\alpha_{n}$ bounded away from 1, $A_{n}$ surely satisfies (A1) for large n.

The mapping $m_{\delta ,\delta^{\prime }}\left(t\right):=\left(d/dt\right)\varphi_{\delta ,\delta^{\prime }}\left(t\right)$ is a homeomorphism from $N_{\delta ,\delta^{\prime }}$ onto the closure of the convex hull of the support of the distribution of $Z_{\delta^{\prime }}$ under $F_{\delta }$ (see, e.g., [14]). Denote

$$\underset{\delta }{ess sup}Z_{\delta^{\prime }}:=\sup\left\{x:\mathrm{for}\;\mathrm{all}\;\epsilon >0,\;F_{\delta }\left(Z_{\delta^{\prime }}\in \left(x-\epsilon ,x\right)>0\right)\right\}.$$

We assume that

(A2) $$\underset{\delta }{ess sup}Z_{\delta^{\prime }}=+\infty ,$$

which is convenient for our task, and quite common in practical industrial modelling. This assumption may be weakened, at notational cost mostly. It follows that

$$\lim_{t\to t^{+}\left(N_{\delta ,\delta^{\prime }}\right)}m_{\delta ,\delta^{\prime }}\left(t\right)=+\infty .$$

It holds that

$$J_{\delta ,\delta^{\prime }}\left(E_{F_{\delta }}\left(Z_{\delta^{\prime }}\right)\right)=0,$$

and, as seen previously

$$\lim_{x\to \infty }J_{\delta ,\delta^{\prime }}\left(x\right)=+\infty .$$

On the other hand,

$$m_{\delta ,\delta^{\prime }}\left(0\right)=E_{F_{\delta }}\left(Z_{\delta^{\prime }}\right)=K\left(F_{\delta },F_{\delta^{\prime }}\right)-K\left(F_{\delta },G_{\delta^{\prime }}\right)<0.$$

Let

$$\begin{array}{cc} I & :=\left(\underset{\delta ,\delta^{\prime }}{\sup}E_{F_{\delta }}\left(Z_{\delta^{\prime }}\right),\infty \right)\\ \; & =\left(\underset{\delta ,\delta^{\prime }}{\sup}K\left(F_{\delta },F_{\delta^{\prime }}\right)-K\left(F_{\delta },G_{\delta^{\prime }}\right),\infty \right). \end{array}$$

By (A2), the interval I is not void.

We now define $A_{n}$ such that (4) holds, namely

$$P_{H0}\left(H1\right)\leq p_{n}\max_{\delta }\max_{\delta^{\prime }}F_{\delta }\left(T_{n,\delta^{\prime }}>A_{n}\right)\leq \alpha_{n}$$

holds for any $\alpha_{n}$ in $\left(0,1\right).$ Note that

(A3) $$A_{n}\geq \max_{\delta ,\delta^{\prime }}E_{F_{\delta }}\left(Z_{\delta^{\prime }}\right)=\max_{\left(\delta ,\delta^{\prime }\right)\in \Delta \times \Delta }K\left(F_{\delta },F_{\delta^{\prime }}\right)-K\left(F_{\delta },G_{\delta^{\prime }}\right),$$

for all *n* large enough, since $\alpha_{n}$ is bounded away from 1.

The function

$$J\left(x\right):=\min_{\left(\delta ,\delta^{\prime }\right)\in \Delta \times \Delta }J_{\delta ,\delta^{\prime }}\left(x\right)$$

is continuous and increasing, as it is the infimum of a finite collection of continuous increasing functions defined on I.

Since

$$P_{H0}\left(H1\right)\leq p_{n}\exp\left(-nJ(A_{n})\right),$$

given $\alpha_{n}$, define

(A4) $$A_{n}:=J^{-1}\left(-\frac{1}{n}\log\frac{\alpha_{n}}{p_{n}}\right).$$

This is well defined for $\alpha_{n}\in \left(0,1\right)$, as $\sup_{\left(\delta ,\delta^{\prime }\right)\in \Delta \times \Delta }E_{F_{\delta }}\left(Z_{\delta^{\prime }}\right)<0$ and $-\left(1/n\right)\log\left(\alpha_{n}/p_{n}\right)>0.$

### Appendix A.2. The Power Function

We now evaluate a lower bound for the power of this test, making use of the Chernoff inequality to get an upper bound for the second risk.

Starting from (5),

$$P_{H_{1}}\left(H0\right)\leq \underset{\eta \in \Delta }{\sup}G_{\eta }\left(T_{n,\eta }\leq A_{n}\right),$$

and define

$$W_{\eta }:=-\log\frac{g_{\eta }}{f_{\eta }}\left(x\right).$$

It holds that

$$E_{G_{\eta }}\left(W_{\eta }\right)=\int \log\frac{f_{\eta }\left(x\right)}{g_{\eta }\left(x\right)}g_{\eta }\left(x\right)dx=-K\left(G_{\eta },F_{\eta }\right),$$

and

$$m_{\eta }\left(t\right):=\left(d/dt\right)\log E_{G_{\eta }}\left(\exp tW_{\eta }\right),$$

which is an increasing homeomorphism from $M_{\eta }$ onto the closure of the convex hull of the support of $W_{\eta }$ under $G_{\eta }.$ For any η, the mapping

$$x\mapsto I_{\eta }\left(x\right)$$

is a strictly increasing function of $K_{\eta }:=\left(E_{G_{\eta }}\left(W_{\eta }\right),\infty \right)$ onto $\left(0,+\infty \right)$, where the same notation as above holds for ${ess sup}_{\eta }W_{\eta }$ (here under $G_{\eta }$), and where we assumed

(A5) $$\underset{\eta }{ess sup}W_{\eta }=\infty$$

for all η.

Assume that $A_{n}$ satisfies

(A6) $$A_{n}\in K:=\underset{\eta \in \Delta }{⋂}K_{\eta }$$

namely

(A7) $$A_{n}\geq \underset{\eta \in \Delta }{\sup}E_{G_{\eta }}\left(W_{\eta }\right)=-\underset{\eta \in \Delta }{\inf}K\left(G_{\eta },F_{\eta }\right).$$

Making use of the Chernoff inequality, we get

$$P_{H_{1}}\left(H0\right)\leq \exp\left(-n\underset{\eta \in \Delta }{\inf}I_{\eta }\left(A_{n}\right)\right).$$

Each function $x\mapsto I_{\eta }\left(x\right)$ is increasing on $(E_{G_{\eta }}\left(W_{\eta }\right),\infty ).$ Therefore the function

$$x\mapsto I\left(x\right):=\underset{\eta \in \Delta }{\inf}I_{\eta }\left(x\right)$$

is continuous and increasing, as it is the infimum of a finite number of continuous increasing functions on the same interval K, which is not void due to (A5).

We have proven that, whenever (A7) holds, a lower bound tor the test of H0 vs. H1 is given by

(A8) $$\begin{array}{cc} P_{H_{1}}\left(H1\right) & \geq 1-\exp\left(-nI(A_{n})\right)\\ \; & =1-\exp\left(-nI\left(J^{-1}\left(-\frac{1}{n}\log\frac{\alpha_{n}}{p_{n}}\right)\right)\right). \end{array}$$

We now collect the above discussion, in order to complete the proof.

### Appendix A.3. A Synthetic Result

The function *J* is one-to-one from *I* onto $K:=\left(J\left(\sup_{\left(\delta ,\delta^{\prime }\right)\in \Delta \times \Delta }E_{\delta }\left(Z_{\delta^{\prime }}\right)\right),\infty \right).$ Since $F_{\delta },J_{\delta ,\delta^{\prime }}\left(E_{\delta }\left(Z_{\delta^{\prime }}\right)\right)$ = 0, it follows that $J\left(\sup_{\left(\delta ,\delta^{\prime }\right)\in \Delta \times \Delta }E_{\delta }\left(Z_{\delta^{\prime }}\right)\right)\geq 0.$ Since $E_{F_{\delta }}\left(Z_{\delta^{\prime }}\right)=K\left(F_{\delta },F_{\delta^{\prime }}\right)-K\left(F_{\delta },G_{\delta^{\prime }}\right)<0$, whenever $\alpha_{n}$ in $\left(0,1\right)$ there exists a unique $A_{n}\in \left(-\inf_{\left(\delta ,\delta^{\prime }\right)\in \Delta \times \Delta }\left(K\left(F_{\delta },G_{\delta^{\prime }}\right)-K\left(F_{\delta },F_{\delta^{\prime }}\right)\right),\infty \right)$ which defines the critical region with level $\alpha_{n}.$

For the lower bound on the power of the test, we have assumed $A_{n}\in K=\left(\sup_{\eta \in \Delta }E_{\eta }\left(W_{\eta }\right),\infty \right)=\left(-\inf_{\eta \in \Delta }K\left(G_{\eta },F_{\eta }\right),\infty \right)$.

In order to collect our results in a unified setting, it is useful to state some connection between $\inf_{\left(\delta ,\delta^{\prime }\right)\in \Delta \times \Delta }\left[K\left(F_{\delta },G_{\delta^{\prime }}\right)-K\left(F_{\delta },F_{\delta^{\prime }}\right)\right]$ and $\inf_{\eta \in \Delta }K\left(G_{\eta },F_{\eta }\right).$ See (A3) and (A7).

Since $K(G_{\delta },F_{\delta })$ is positive, it follows from (6) that

(A9) $$\underset{\left(\delta ,\delta^{\prime }\right)\in \Delta \times \Delta }{\sup}\int \log\frac{f_{\delta^{\prime }}}{g_{\delta^{\prime }}}f_{\delta }<\underset{\delta \in \Delta }{\sup}K\left(G_{\delta },F_{\delta }\right),$$

which implies the following fact:

Let $\alpha_{n}$ be bounded away from 1. Then (A3) is fulfilled for large *n*, and therefore there exists $A_{n}$ such that

$$\underset{\delta \in \Delta }{\sup}F_{\delta }\left(T_{n}>A_{n}\right)\leq \alpha_{n}.$$

Furthermore, by (A9), Condition (A7) holds, which yields the lower bound for the power of this test, as stated in (A8).

## Appendix B. Proof of Theorem 3

We will repeatedly make use of the following result (Theorem 3 in [15]), which is an extension of the Chernoff-Stein Lemma (see [16]).

**Theorem** **A1.**
*[Krafft and Plachky] Let $x_{n}$, such that*

$$F_{\delta }\left(T_{n,\delta }>x_{n}\right)\leq \alpha_{n}$$

*with $limsup_{n\to \infty }\alpha_{n}<1.$ Then*

$$\lim_{n\to \infty }\frac{1}{n}\log G_{\delta }\left(T_{n,\delta }\leq x_{n}\right)=-K\left(F_{\delta },G_{\delta }\right).$$

**Remark** **A2.**
*The above result indicates that the power of the Neyman-Pearson test only depends on its level on the second order on the logarithmic scale.*

Define $A_{n,\delta_{*}}$ such that

$$F_{\delta_{*}}\left(T_{n}\leq A_{n}\right)=F_{\delta_{*}}\left(T_{n,\delta_{*}}\leq A_{n,\delta_{*}}\right).$$

This exists and is uniquely defined, due to the regularity of the distribution of $T_{n,\delta_{*}}$ under $F_{\delta_{*}}.$ Since $1\left[T_{n,\delta_{*}}>A_{n}\right]$ is the likelihood ratio test of $H_{0}\left(\delta_{*}\right)$ against $H_{1}\left(\delta_{*}\right)$ of the size $\alpha_{n},$ it follows, by unbiasedness of the LRT, that

$$F_{\delta_{*}}\left(T_{n}\leq A_{n}\right)=F_{\delta_{*}}\left(T_{n,\delta_{*}}\leq A_{n,\delta_{*}}\right)\geq G_{\delta_{*}}\left(T_{n,\delta_{*}}\leq A_{n,\delta_{*}}\right).$$

We shall later verify the validity of the conditions of Theorem A1; namely, that

(A10) $$\lim\underset{n\to \infty }{\sup}F_{\delta_{*}}\left(T_{n,\delta_{*}}\leq A_{n,\delta_{*}}\right)<1.$$

Assuming (A10) we get, by Theorem A1,

$$\lim\underset{n\to \infty }{\sup}\frac{1}{n}\log F_{\delta_{*}}\left(T_{n}\leq A_{n}\right)\geq \lim_{n\to \infty }\frac{1}{n}\log G_{\delta_{*}}\left(T_{n,\delta_{*}}\leq A_{n,\delta_{*}}\right)=-K\left(F_{\delta_{*}},G_{\delta_{*}}\right).$$

We shall now prove that

$$\lim_{n\to \infty }\frac{1}{n}\log G_{\delta_{*}}\left(T_{n,\delta_{*}}\leq A_{n,\delta_{*}}\right)=\lim_{n\to \infty }\frac{1}{n}\log G_{\delta_{*}}\left(T_{n}\leq A_{n}\right).$$

Let $B_{n,\delta_{*}}$, such that

$$G_{\delta_{*}}\left(T_{n,\delta_{*}}\leq B_{n,\delta_{*}}\right)=G_{\delta_{*}}\left(T_{n}\leq A_{n}\right).$$

By regularity of the distribution of $T_{n,\delta_{*}}$ under $G_{\delta_{*}}$, such a $B_{n,\delta_{*}}$ is defined in a unique way. We will prove that the condition in Theorem A1 holds, namely

(A11) $$\lim\underset{n\to \infty }{\sup}F_{\delta_{*}}\left(T_{n,\delta_{*}}\leq B_{n,\delta_{*}}\right)<1.$$

$$\lim_{n\to \infty }\frac{1}{n}\log G_{\delta_{*}}\left(T_{n,\delta_{*}}\leq A_{n,\delta_{*}}\right)=\lim_{n\to \infty }\frac{1}{n}\log G_{\delta_{*}}\left(T_{n}\leq A_{n}\right)=-K\left(F_{\delta_{*}},G_{\delta_{*}}\right).$$

Incidentally, we have obtained that $\lim_{n\to \infty }\frac{1}{n}\log G_{\delta_{*}}\left(T_{n}\leq A_{n}\right)$ exists. Therefore we have proven that

$$\lim\underset{n\to \infty }{\sup}\frac{1}{n}\log F_{\delta_{*}}\left(T_{n}\leq A_{n}\right)\geq \lim_{n\to \infty }\frac{1}{n}\log G_{\delta_{*}}\left(T_{n}\leq A_{n}\right),$$

which is a form of unbiasedness. For $\delta \neq \delta_{*}$, let $B_{n,\delta }$ be defined by

$$G_{\delta }\left(T_{n,\delta }\leq B_{n,\delta }\right)=G_{\delta }\left(T_{n}\leq A_{n}\right).$$

As above, $B_{n,\delta }$ is well-defined. Assuming

(A12) $$\lim\underset{n\to \infty }{\sup}F_{\delta }\left(T_{n,\delta }\leq B_{n,\delta }\right)<1,$$

it follows, from Theorem A1, that

$$\lim_{n\to \infty }\frac{1}{n}\log G_{\delta }\left(T_{n}\leq A_{n}\right)=\lim_{n\to \infty }\frac{1}{n}\log G_{\delta }\left(T_{n,\delta }\leq B_{n,\delta }\right)=-K\left(F_{\delta },G_{\delta }\right).$$

Since $K\left(F_{\delta_{*}},G_{\delta_{*}}\right)\leq K\left(F_{\delta },G_{\delta }\right),$ we have proven

$$\lim\underset{n\to \infty }{\sup}\frac{1}{n}\log F_{\delta_{*}}\left(T_{n}\leq A_{n}\right)\geq \lim_{n\to \infty }\frac{1}{n}\log G_{\delta_{*}}\left(T_{n}\leq A_{n}\right)\geq \lim_{n\to \infty }\frac{1}{n}\log G_{\delta }\left(T_{n}\leq A_{n}\right).$$

It remains to verify the conditions (A10)–(A12). We will only verify (A12), as the two other conditions differ only by notation. We have

$$\begin{array}{cc} G_{\delta }\left(T_{n,\delta }>B_{n,\delta }\right) & =G_{\delta }\left(T_{n}>A_{n}\right)\leq F_{\delta }\left(T_{n}>A_{n}\right)+d_{TV}\left(F_{\delta },G_{\delta }\right)\\ \; & \leq \alpha_{n}+d_{TV}\left(F_{\delta },G_{\delta }\right)<1, \end{array}$$

by hypothesis (13). By the law of large numbers, under $G_{\delta }$

$$\lim_{n\to \infty }T_{n,\delta }=K\left(G_{\delta },F_{\delta }\right)\;\left[G_{\delta }-\mathrm{a.s.}\right].$$

Therefore, for large *n*,

$$\lim\underset{n\to \infty }{\inf}B_{n,\delta }\geq K\left(G_{\delta },F_{\delta }\right)\;\left[G_{\delta }-\mathrm{a.s.}\right].$$

Since, under $F_{\delta }$,

$$\lim_{n\to \infty }T_{n,\delta }=-K\left(F_{\delta },G_{\delta }\right)\;\left[F_{\delta }-\mathrm{a.s.}\right],$$

this implies that

$$\lim_{n\to \infty }F_{\delta }\left(T_{n,\delta }>B_{n,\delta }\right)<1.$$

## Appendix C. Proof of Proposition 4

We now prove the three lemmas that we used.

**Lemma** **A3.**
*Let P, Q, and R denote three distributions with respective continuous and bounded densities p, q, and r. Then*

(A13) K(P∗R,Q∗R)≤K(P,Q).

**Proof.** Let $P:=\left(A_{1},\ldots ,A_{K}\right)$ be a partition of R and $p:=\left(p_{1},\ldots ,p_{K}\right)$ denote the probabilities of $A_{1},\ldots ,A_{K}$ under P. Set the same definition for $q_{1},\ldots ,q_{K}$ and for $r_{1},\ldots ,r_{K}.$ Recall that the log-sum inequality writes

$$\left(\sum a_{i}\right)\log\frac{\sum b_{i}}{\sum c_{i}}\leq \sum a_{i}\log\frac{b_{i}}{c_{i}}$$

for positive vectors ${\left(a_{i}\right)}_{i}$, ${\left(b_{i}\right)}_{i}$ and ${\left(c_{i}\right)}_{i}.$ By the above inequality, for any i∈{1,…,K}, denoting $\left(p*r\right)$ to be the convolution of p and r,

$${\left(p*r\right)}_{j}\log\frac{{\left(p*r\right)}_{j}}{{\left(q*r\right)}_{j}}\leq \sum_{i=1}^{K}p_{j}r_{i-j}\log\frac{p_{j}r_{i-j}}{q_{j}r_{i-j}}.$$

Summing over j∈{1,…,K} yields

$$\sum_{j=1}^{K}{\left(p*r\right)}_{j}\log\frac{{\left(p*r\right)}_{j}}{{\left(q*r\right)}_{j}}\leq \sum_{j=1}^{K}p_{j}\log\frac{p_{j}}{q_{j}},$$

which is equivalent to

$$K_{P}\left(P*R,Q*R\right)\leq K_{P}\left(P,Q\right),$$

where $K_{P}$ designates the Kullback-Leibler divergence defined on P. Refine the partition and go to the limit (Riemann Integrals), to obtain (A13). □

We now set a classical general result which states that, when $R_{\delta }$ denotes a family of distributions with some decomposability property, then the Kullback-Leibler divergence between $P*R_{\delta }$ and $Q*R_{\delta }$ is a decreasing function of δ.

**Lemma** **A4.**
*Let P and Q satisfy the hypotheses of Lemma A3 and let ${\left(R_{\delta }\right)}_{\delta >0}$ denote a family of p.m.’s on R, and denote accordingly $V_{\delta }$ to be a r.v. with distribution $R_{\delta }.$ Assume that, for all δ and η, there exists a r.v. $W_{\delta ,\eta }$, independent upon $V_{\delta }$, such that*

$$V_{\delta +\eta }{=}_{d}V_{\delta }+W_{\delta ,\eta }.$$

*Then the function $\delta \mapsto K\left(P*R_{\delta },Q*R_{\delta }\right)$ is non-increasing.*

**Proof.** Using Lemma A3, it holds that, for positive *η*,

$$\begin{array}{cc} K\left(P*R_{\delta +\eta },Q*R_{\delta +\eta }\right) & =K\left(\left(P*R_{\delta }\right)*W_{\delta ,\eta },\left(Q*R_{\delta }\right)*W_{\delta ,\eta }\right)\\ \; & \leq K\left(P*R_{\delta },Q*R_{\delta }\right), \end{array}$$

which proves the claim. □

**Lemma** **A5.**
*Let P, Q, and R be three probability distributions with respective continuous and bounded densities p,q, and r.Assume that*

K(P,Q)≤K(Q,P),

*where all involved quantities are assumed to be finite. Then*

K(P∗R,Q∗R)≤K(Q∗R,P∗R).

**Proof.** We proceed as in Lemma A3, using partitions and denoting by $p_{1},\ldots ,p_{K}$ the induced probability of P on P. Then,

$$\begin{array}{cc} K_{P}\left(P*R,Q*R\right)-K_{P}\left(Q*R,P*R\right) & =\sum_{i}\sum_{j}\left(p_{j}r_{i-j}+q_{j}r_{i-j}\right)\log\frac{\sum_{j}p_{j}r_{i-j}}{\sum_{j}q_{j}r_{i-j}}\\ \; & \leq \sum_{j}\sum_{i}\left(p_{j}r_{i-j}+q_{j}r_{i-j}\right)\log\frac{p_{j}}{q_{j}}\\ \; & =\sum_{j}\left(p_{j}+q_{j}\right)\log\frac{p_{j}}{q_{j}}\\ \; & =K_{P}\left(P,Q\right)-K_{P}\left(Q,P\right)\leq 0, \end{array}$$

where we used the log-sum inequality and the fact that K(P,Q)≤K(Q,P) implies $K_{P}\left(P,Q\right)\leq K_{P}\left(Q,P\right)$, by the data-processing inequality. □
